# Supplementary material for: Low-Rank Tensor Encoding Models Decompose Natural Speech Comprehension Processes
Source: bioRxiv. 2025 Jun 3:2025.06.02.657514. Preprint. [Version 1] doi: 10.1101/2025.06.02.657514 (PMC12157526; doi:10.1101/2025.06.02.657514)
Supplement: Supplement 1 [file media-1.pdf]

Supplemental Material

May 22, 2025

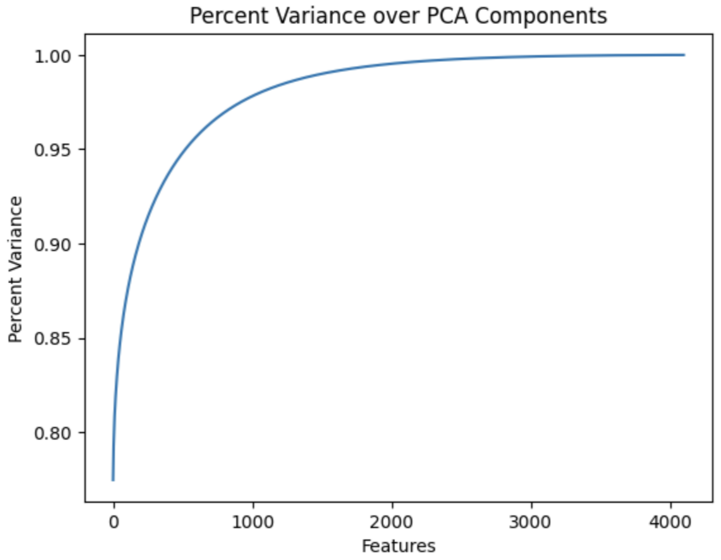

Figure 1: Embedding percent variance explained over PCA components of Llama2-7b on the Moth Radio Corpus. 95% of the variance is captured at a small percent of the total dimension.

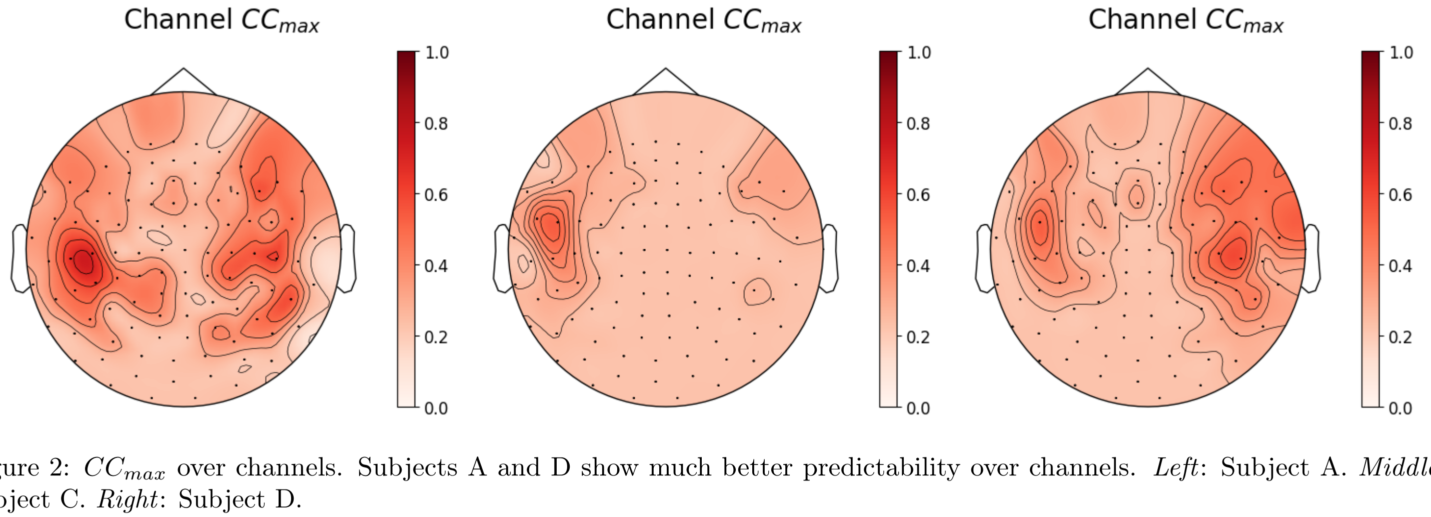

Figure 2: CC_{max} over channels. Subjects A and D show much better predictability over channels. *Left*: Subject A. *Middle*: Subject C. *Right*: Subject D.

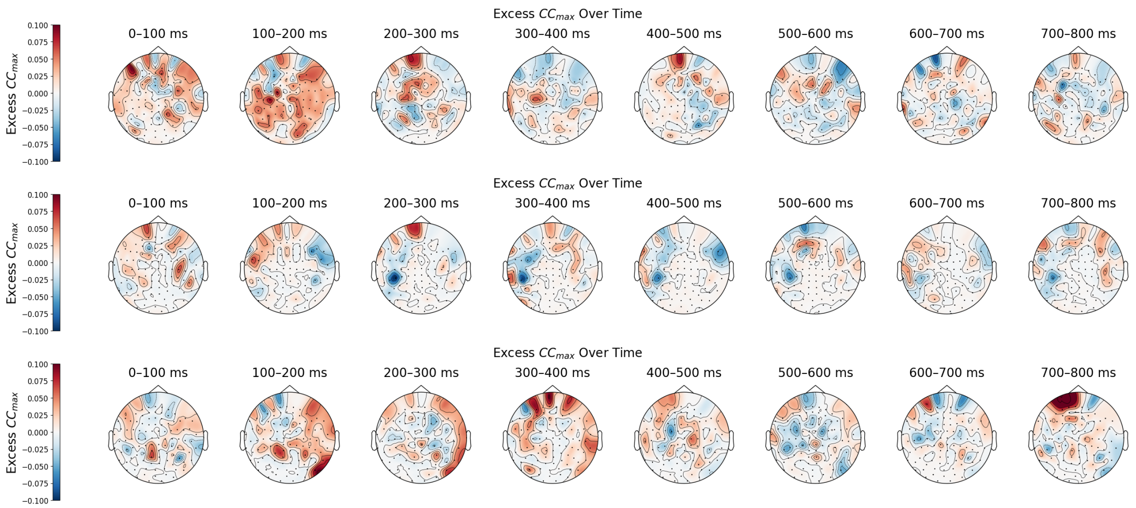

Figure 3: Channel excess CC_{max} shows distinct patterns over time. Subjects A and D show early peaks in predictability around 100-200ms post word onset and an additional shallow peak at 400-500ms in subject A and 300-400ms in subject D. *Upper panel*: Subject A. *Middle panel*: Subject C. *Lower panel*: Subject D.

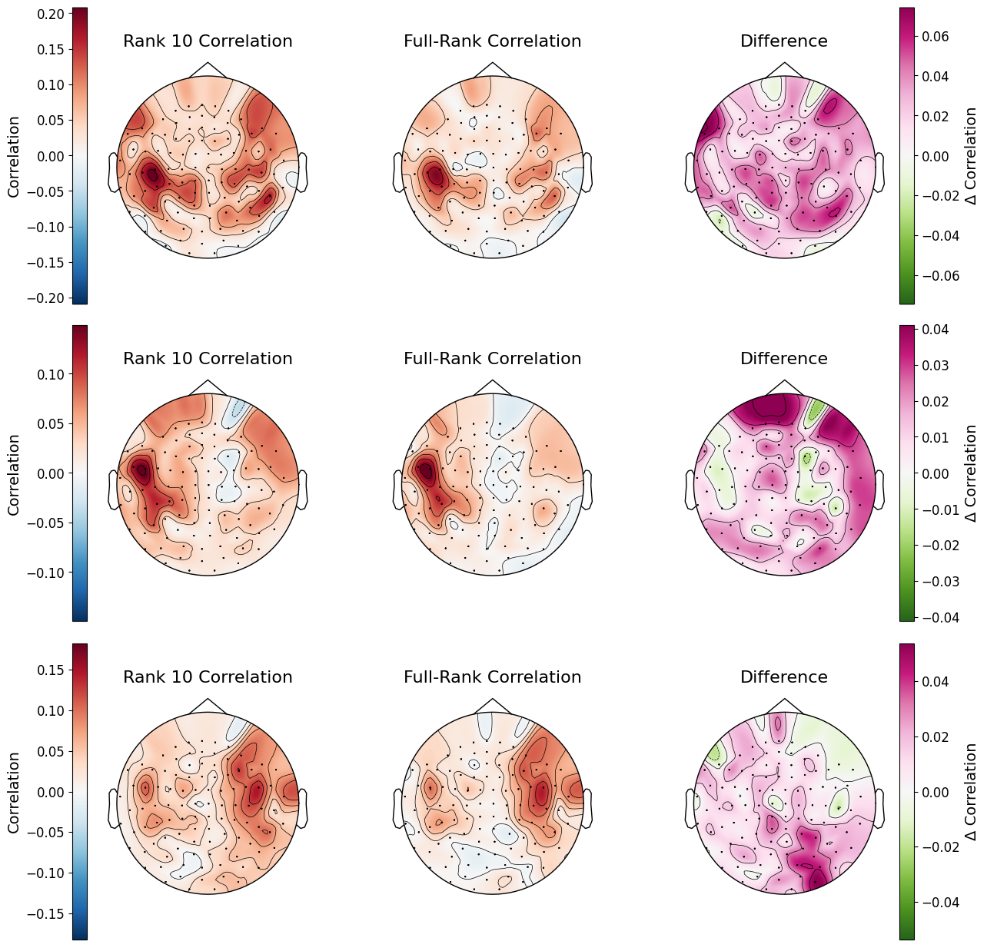

Figure 4: Rank 10 models show widespread performance improvements over full ridge regression. *Top panel*: Subject A. *Middle panel*: Subject C. *Bottom panel*: Subject D.

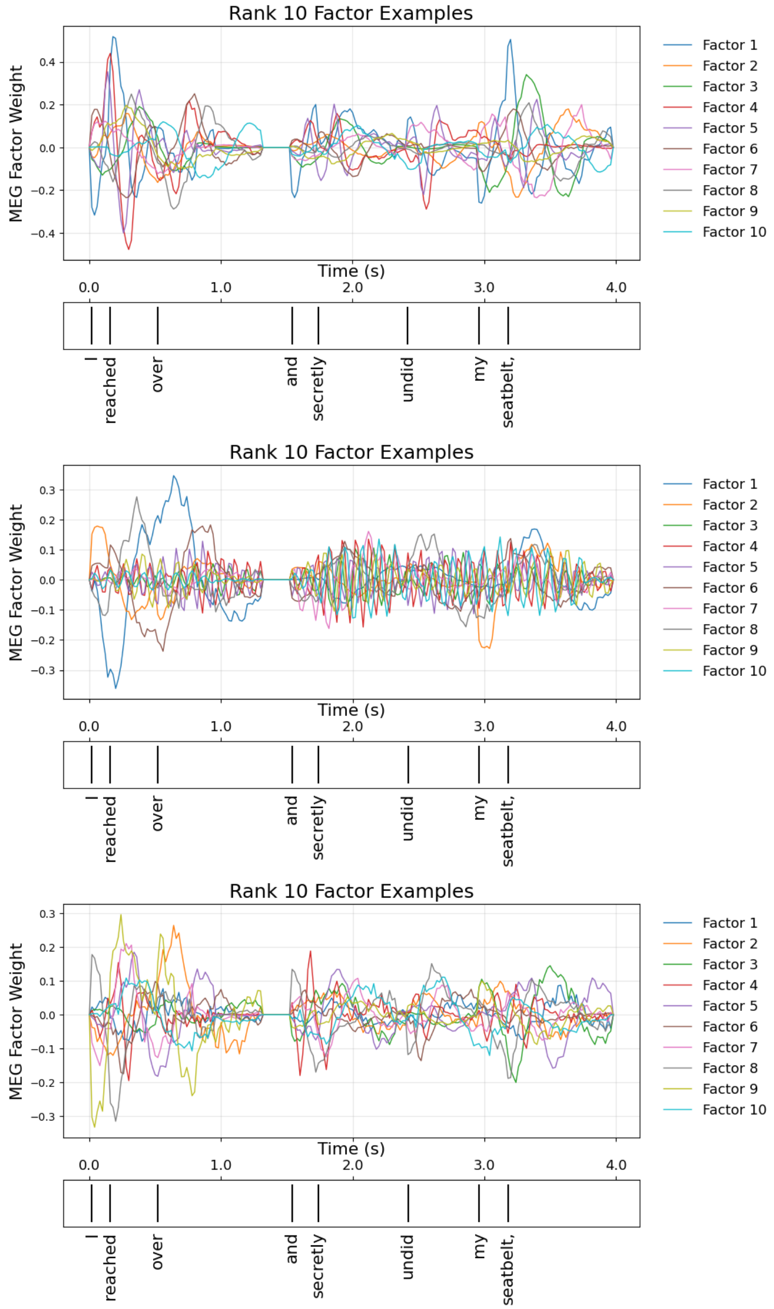

Figure 5: Timecourses of components pre-MEG factor multiplication. Factors show a diversity of timecourses in response to natural language. *Top panel*: Subject A. *Middle panel*: Subject C. Note the very fast components, likely corresponding to the small amount of signal in subject C compared to the number of ranks. *Bottom panel*: Subject D.

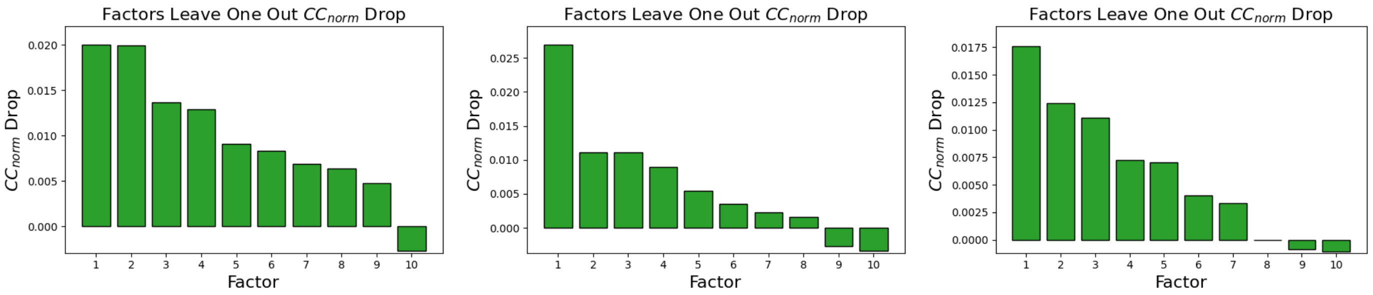

Figure 6: Leave one component out drop in CC_{norm} over rank. Components show different influence on test performance. *Left*: Subject A. *Middle*: Subject C. *Right*: Subject D.

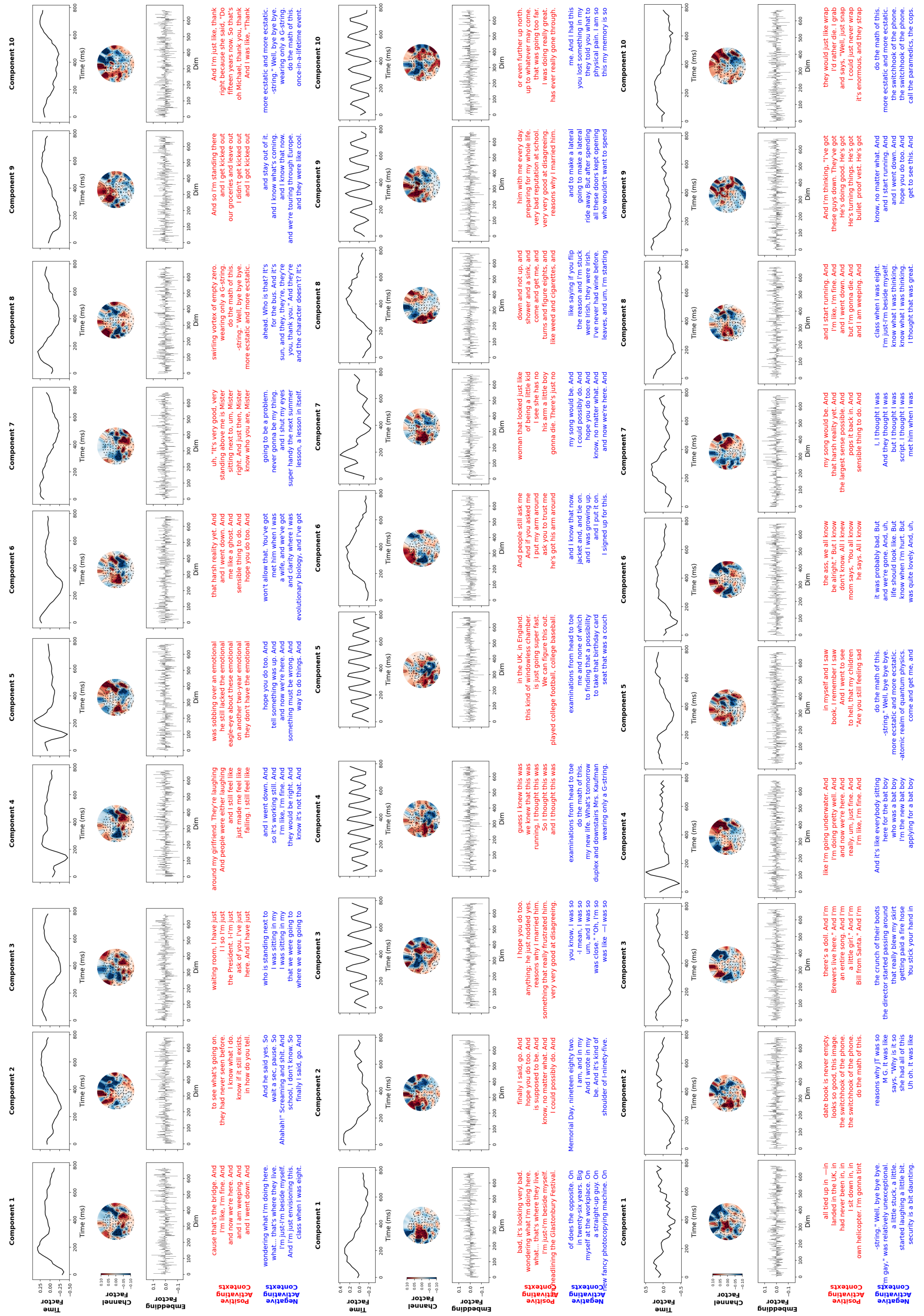

Figure 7: Subjects show a diversity of timecourses, spatial components, and activating texts. *Left panel*: Subject A. *Middle panel*: Subject C. Note the fast timecourse components, likely stemming from low signal in the subject. *Right panel*: Subject D.

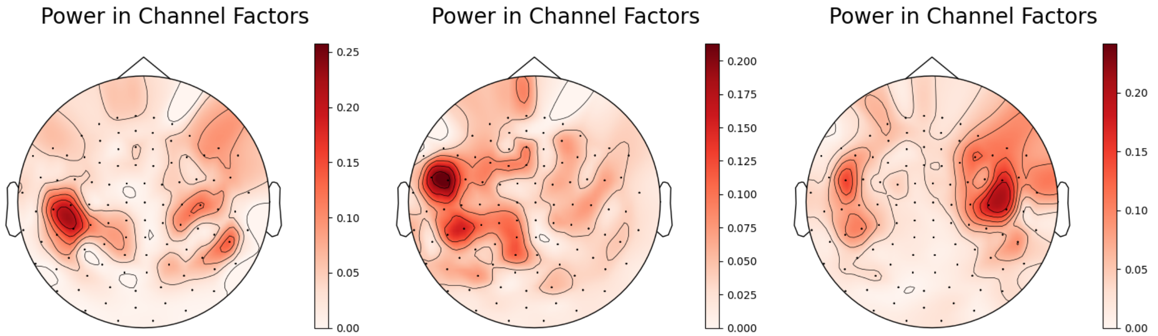

Figure 8: Using the same power analysis as main figure 5 shows that subject channel factors concentrate over auditory areas (left and right hemisphere, roughly in temporal and prefrontal regions). *Left*: Subject A. *Middle*: Subject C. *Right*: Subject D.

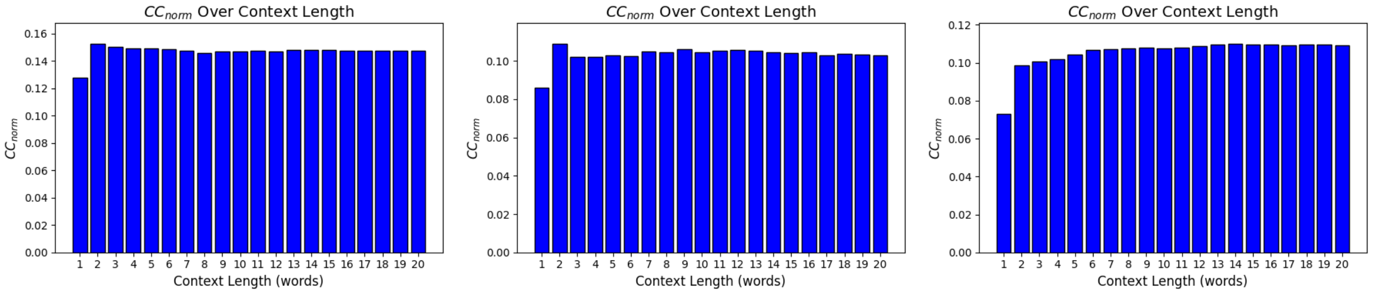

Figure 9: CC_{norm} saturates after a small number of previous words being included in the context when evaluated on a rank-10 model trained on a 20 word context window. *Left*: Subject A. *Middle*: Subject C. *Right*: Subject D.

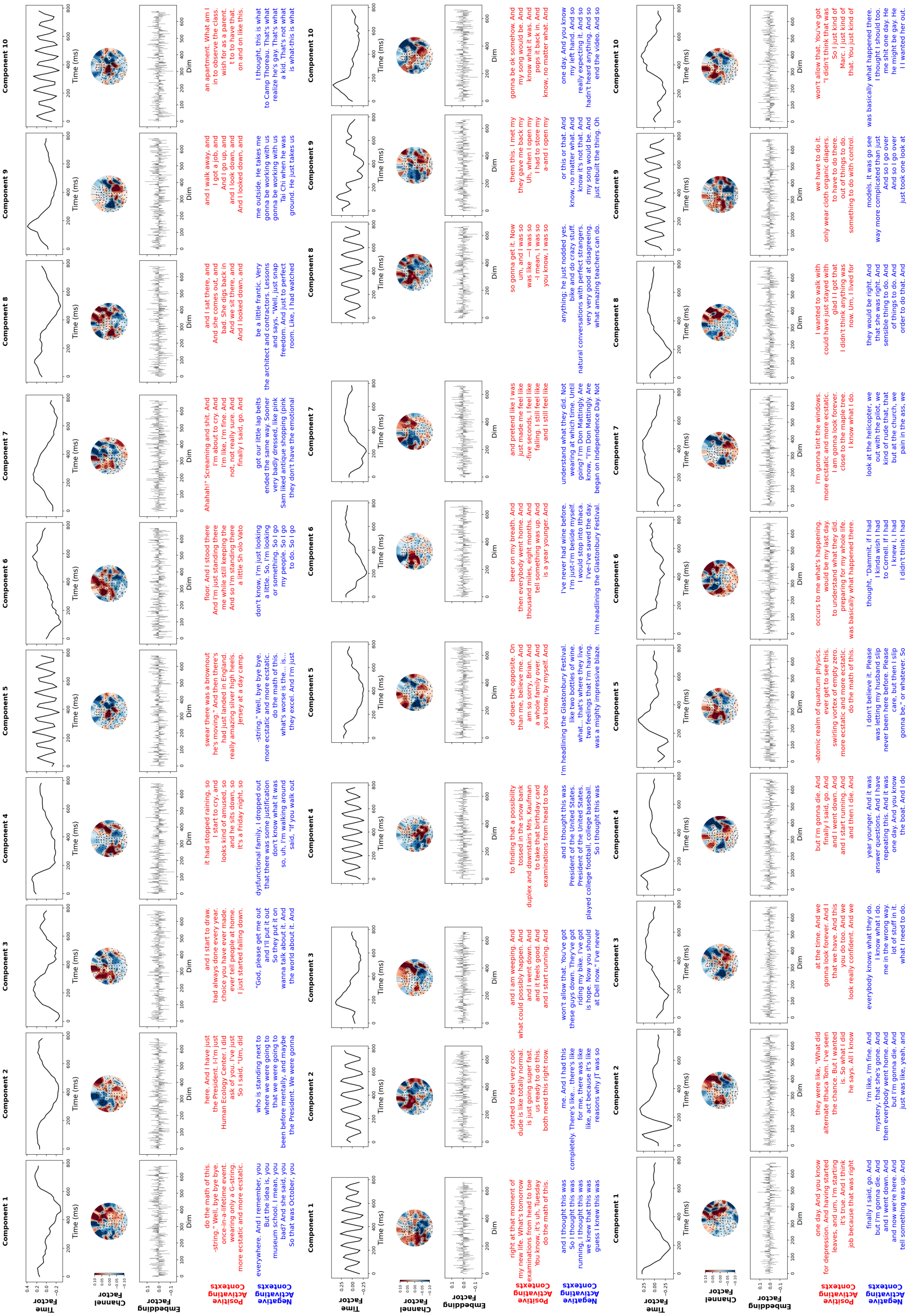

Figure 10: Control subtracted factors for a rank-10 model. We see changes in timecourses, channels and activating texts from the un-controlled rank-10 model. Note the fast timecourses, likely arising from less signal in control subtracted residuals. *Left panel*: Subject A. *Middle panel*: Subject C. *Right panel*: Subject D.

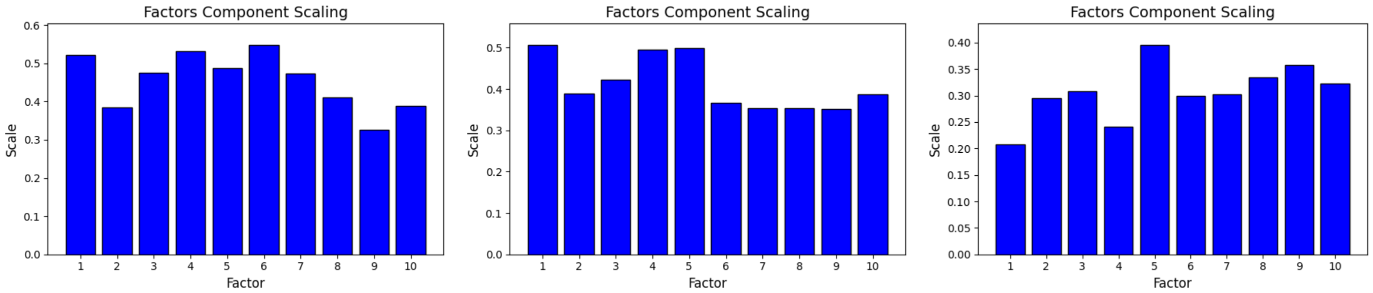

(a) Component scalings show roughly similar ranges over components despite having different influences on test performance (see Supplemental Figure 6).*Left*: Subject A. *Middle*: Subject C. *Right*: Subject D.

Subject	Rank 1	Rank 2	Rank 3	Rank 4	Rank 5	Rank 6	Rank 7	Rank 8	Rank 9	Rank 10
A	0.001	0.001	0.001	0.001	0.001	0.001	0.001	0.001	0.001	0.001
C	0.208	0.001	0.001	0.001	0.001	0.001	0.001	0.001	0.001	0.001
D	0.783	0.001	0.001	0.001	0.001	0.001	0.001	0.001	0.001	0.001

Subject	Rank 11	Rank 12	Rank 13	Rank 14	Rank 15	Rank 16	Rank 17	Rank 18	Rank 19	Rank 20
A	0.001	0.001	0.001	0.001	0.001	0.001	0.001	0.001	0.001	0.001
C	0.001	0.001	0.001	0.001	0.001	0.001	0.001	0.001	0.001	0.001
D	0.001	0.001	0.001	0.001	0.001	0.001	0.001	0.001	0.001	0.001

(b) CC_{norm} differences between low-rank models vs full linear rank regression are significant at 0.001 for all models except for Subject C and D rank-1 models. Shown above are the estimated p-values from a bootstrapped test performance measure. To estimate the p-value, test data was split into sequential blocks of 10 seconds, sampled from with replacement and used to construct a new test dataset. The performance difference between the low-rank model and full rank model were calculated for each new test set. The p-value is then the ratio of performances less than 0 to total bootstraps, in this case 1000

Figure 11: Component scaling over factors and significance difference tests between rank models and full rank ridge regression.

Subject	Rank 1	Rank 2	Rank 3	Rank 4	Rank 5	Rank 6	Rank 7	Rank 8	Rank 9	Rank 10
A	0.001	0.001	0.001	0.001	0.001	0.001	0.001	0.001	0.001	0.001
C	0.001	0.001	0.001	0.001	0.001	0.001	0.001	0.001	0.001	0.001
D	0.001	0.001	0.001	0.001	0.001	0.001	0.001	0.001	0.001	0.001

Subject	Rank 11	Rank 12	Rank 13	Rank 14	Rank 15	Rank 16	Rank 17	Rank 18	Rank 19	Rank 20
A	0.001	0.001	0.001	0.001	0.001	0.001	0.001	0.001	0.001	0.001
C	0.001	0.001	0.001	0.001	0.001	0.001	0.001	0.001	0.001	0.001
D	0.001	0.001	0.001	0.001	0.001	0.001	0.001	0.001	0.001	0.001

Figure 12: CC_{norm} performance is above chance for all models at 0.001. Shown above are the estimated p-values from a permuted test performance measure. To estimate p-values, we first split our test MEG data into sequential blocks of 10 seconds. Then for each permutation sample, we shuffled the blocks to create a new test set and computed the performance of the model on it. Using all permutations (in this case 1000), we construct the null distribution that our model is equal to a chance model. Then, we obtain the p-value as the probability that the null distribution is greater than the test performance of the model.

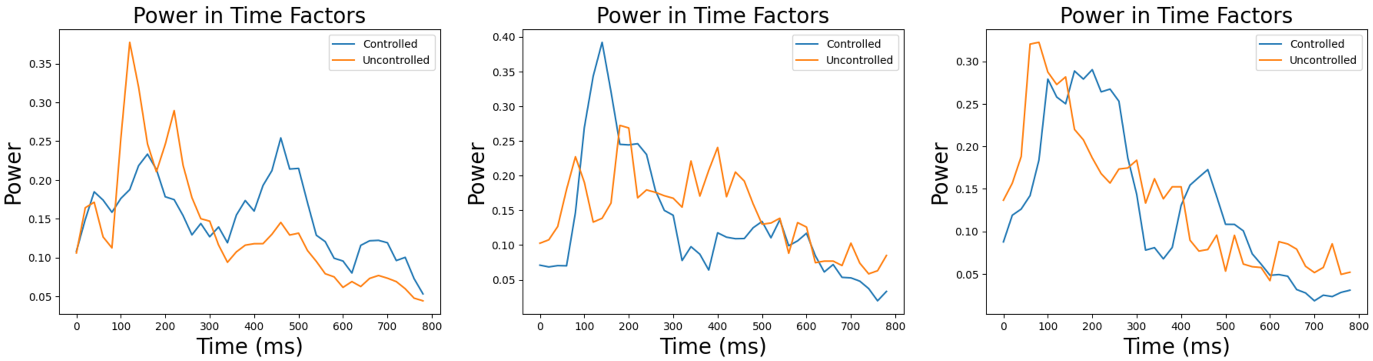

Figure 13: The power in the time factors shifts to be later in subjects A and D after subtracting controls. In subjects A and D, the power is approximately monotonic before subtracting controls, peaking at around 150ms. After control subtraction, double peaks form, one early peak at around 150-200ms followed by a later peak around 400-500ms. Subject C exhibits this double peak behavior, however after controls, it becomes more peaked around 150ms. *Left*: Subject A. *Middle*: Subject C. *Right*: Subject D.

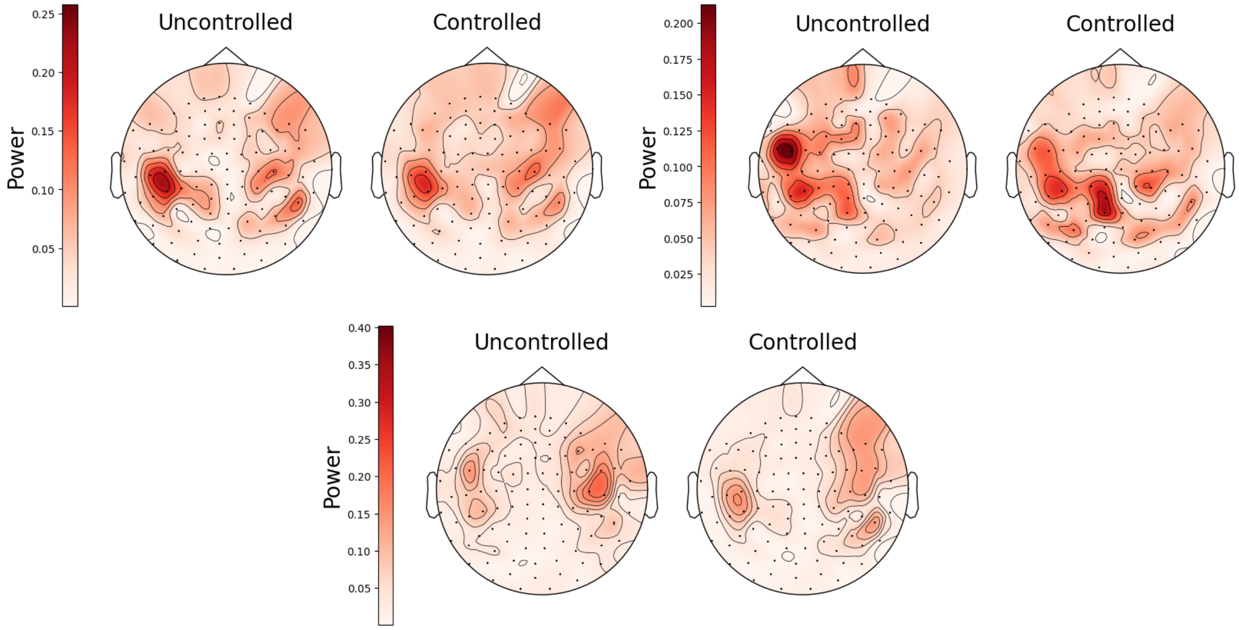

Figure 14: Power in channel factors before and after subtracting controls. Generally, across subjects, channel factors shift to become less concentrated over auditory areas, affecting channels more globally following control subtraction. *Top left*: Subject A. *Top right*: Subject C. *Bottom middle*: Subject D.
